# Vibrational analysis of acetylcholine binding to the M_2_ receptor†

**DOI:** 10.1039/d1ra01030a

**Published:** 2021-04-07

**Authors:** Kohei Suzuki, Kota Katayama, Yuji Sumii, Tomoya Nakagita, Ryoji Suno, Hirokazu Tsujimoto, So Iwata, Takuya Kobayashi, Norio Shibata, Hideki Kandori

**Affiliations:** Department of Life Science and Applied Chemistry, Nagoya Institute of Technology Showa-ku Nagoya 466-8555 Japan kandori@nitech.ac.jp; OptoBioTechnology Research Center, Nagoya Institute of Technology Showa-ku Nagoya 466-8555 Japan; Department of Medical Chemistry, Kansai Medical University Hirakata 573-1010 Japan; Department of Cell Biology, Graduate School of Medicine, Kyoto University Kyoto 606-8501 Japan; Japan Agency for Medical Research and Development, Core Research for Evolutional Science and Technology (AMED-CREST) Tokyo 100-0004 Japan

## Abstract

The M_2_ muscarinic acetylcholine receptor (M_2_R) is a prototypical G protein-coupled receptor (GPCR) that responds to acetylcholine (ACh) and mediates various cellular responses in the nervous system. We recently established Attenuated Total Reflection-Fourier Transform Infrared (ATR-FTIR) spectroscopy for ligand binding to M_2_R reconstituted in lipid membranes, paving the way to understand the mechanism in atomic detail. However, the obtained difference FTIR spectra upon ligand binding contained ligand, protein, lipid, and water signals, so a vibrational assignment was needed for a thorough understanding. In the present study, we compared difference FTIR spectra between unlabeled and 2-^13^C labeled ACh, and assigned the bands at 1741 and 1246 cm^−1^ as the C=O and C–O stretches of ACh, respectively. The C=O stretch of ACh in M_2_R is close to that in aqueous solution (1736 cm^−1^), and much lower in frequency than the free C=O stretch (1778–1794 cm^−1^), indicating a strong hydrogen bond, which probably formed with N404^6.52^. We propose that a water molecule bridges ACh and N404^6.52^. The other ACh terminal is positively charged, and it interacts with negatively charged D103^3.32^. The present study revealed that D103^3.32^ is deprotonated (negatively charged) in both ACh-bound and free states, a suggested mechanism to stabilize the negative charge of D103^3.32^ in the free M_2_R.

## Abstract 2

We recently reported difference FTIR spectra upon binding of Ach to M_2_R.

## Graphical abstract

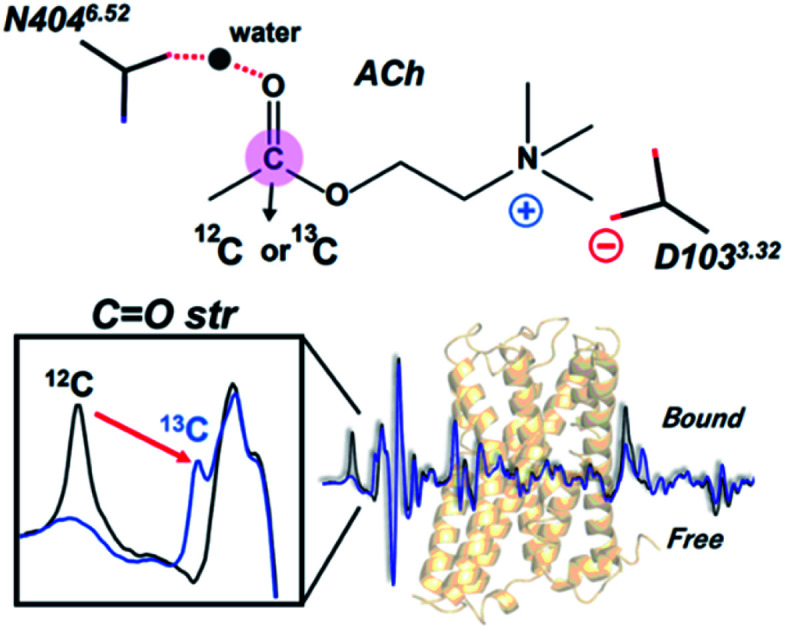

G protein-coupled receptor (GPCR) signaling utilizes a coupling mechanism between the extracellular ligand-binding pocket and the cytoplasmic domain of the receptor that selectively interacts with a signaling transducer.^1,2^ This allosteric effect enables one site of the receptor to regulate the function of another spatially distinct region. Therefore, it is important to understand the molecular mechanism behind selective ligand-induced changes in receptor conformation and specific transducer-recognition for the development of GPCR-targeting drugs. Both X-ray crystallography and cryo-electron microscopy techniques have played important roles in determining structures of inactive and active GPCR states bound to orthosteric and allosteric ligands.^3–5^ In addition, spectroscopic techniques such as NMR and double electron–electron resonance (DEER) of multiple GPCR states have provided valuable information on their dynamic nature.^1,6–9^ More recently, solution NMR was used to structurally link ligand-binding to M_2_ muscarinic acetylcholine receptor (M_2_R) with its G-protein coupling interface.^10^

M_2_R is one of the best studied GPCRs, and its structure and structural dynamics have been reported by X-ray crystallography,^11–13^ cryo-electron microscopy,^14,15^ and NMR.^10^Fig. 1a illustrates the structure of the active state that binds an agonist iperoxo, and highlights the binding site composed of D103^3.32^, N404^6.52^, and a tyrosine lid (Y104^3.33^, Y403^6.51^, and Y426^7.39^). The antagonist binding form of M_2_R was also reported.^11^ While these structures provided useful structural information, no structures have yet been suggested for the binding form of acetylcholine (ACh), the native ligand, and the ligand-free form. Therefore, it is unknown how key residues respond to the binding of ACh. For instance, it is reasonable to postulate that D103^3.32^ is deprotonated in the ACh-binding form to stabilize the positively charged ACh. Yet what is the protonation state of D103^3.32^ in the ligand-free form? And how do N404^6.52^ and tyrosines alter their structures before and after ACh binding? A similar question is equally applicable to ACh itself, namely how is the structure altered upon binding to M_2_R?

**Fig. 1:**
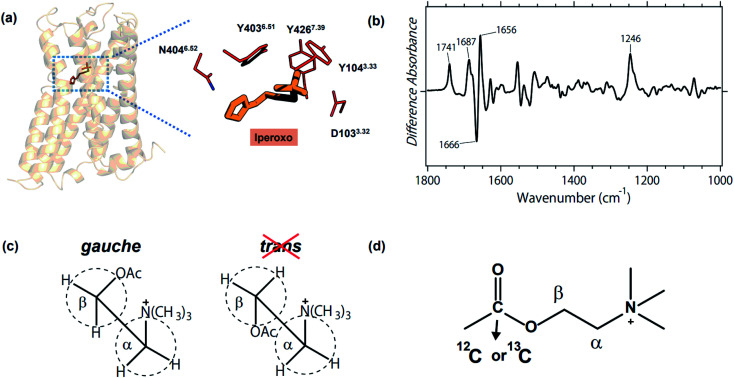
(a) Crystal structure of the active M_2_R in complex with agonist iperoxo (left, PDB: 4MQS), and the enlarged ligand binding site (right). D103^3.32^, N404^6.52^, and the tyrosine lid (Y104^3.33^, Y403^6.51^, and Y426^7.39^) constitute the binding site of iperoxo, whereas the structure of the native ligand, ACh, remains unknown. (b) The acetylcholine binding-induced difference ATR-FTIR spectrum, which is reproduced from ref. 18. (c) Gauche and *trans* forms of ACh. Previous FTIR study of methacholine and acetylthiocholine revealed the gauche form being the conformation of the native ligand.^18^ (d) Chemical structure of ACh and the ^13^C-labeled position in the present study.

Stimulus-induced difference Fourier-transform infrared (FTIR) spectroscopy is a powerful, sensitive and informative tool to investigate protein structural changes that accompany biologically important functional processes.^16,17^ Although light has been the ideal stimulus for difference FTIR spectroscopy, we attempted to extend the application of ligand-binding to GPCR by using Attenuated Total Reflection (ATR)-FTIR spectroscopy. Fig. 1b shows the first difference FTIR spectrum thus obtained for ACh-binding to human M_2_R.^18^ The measurements were performed in membrane lipids, which is more native than in protein crystal (X-ray crystallography) and in detergent (cryo-electron microscopy, NMR, and DEER). A small amount of the sample (5 μg) is another advantage of difference FTIR spectroscopy.

In a previous study, we compared the data for ACh (Fig. 1b) with those for methacholine (gauche-conformation preferable) and acetylthiocholine (*trans*-conformation preferable), concluding that ACh binds with M_2_R in its gauche conformation (Fig. 1c).^18^ Positive and negative bands in Fig. 1b originate from ACh-bound and free M_2_R, respectively, and the bands at 1666 (−)/1656 (+) cm^−1^ indicate a helical structural perturbation upon ACh binding. In addition, positive peaks at 1741, 1687, and 1246 cm^−1^ are possibly due to the vibrations of the carboxylic C=O stretch (D103^3.32^), carbonyl C=O stretch (N404^6.52^), and phenolic C–O stretch (Y104^3.33^, Y403^6.51^, and Y426^7.39^), respectively. It should be noted, however, that the difference FTIR spectra contain signals of ACh, which also possesses C=O and C–O stretches (Fig. 1d). Therefore, we are able to interpret vibrational bands only when they are assigned by using isotope-labeling. In this study, we synthesized 2-^13^C-labeled ACh (Fig. 1d), whose difference FTIR spectra were compared with those of unlabeled ACh. Vibrational assignment of the C=O and C–O stretches of ACh provides structural insight into not only the ligand, but also the protein moiety such as D103^3.32^ and the Tyr lid. The mechanism by which ACh binds to M_2_R will be discussed based on the present FTIR observations.

## Results

### Difference FTIR spectra with 2-^13^C-labeled acetylcholine

**Fig. 2:**
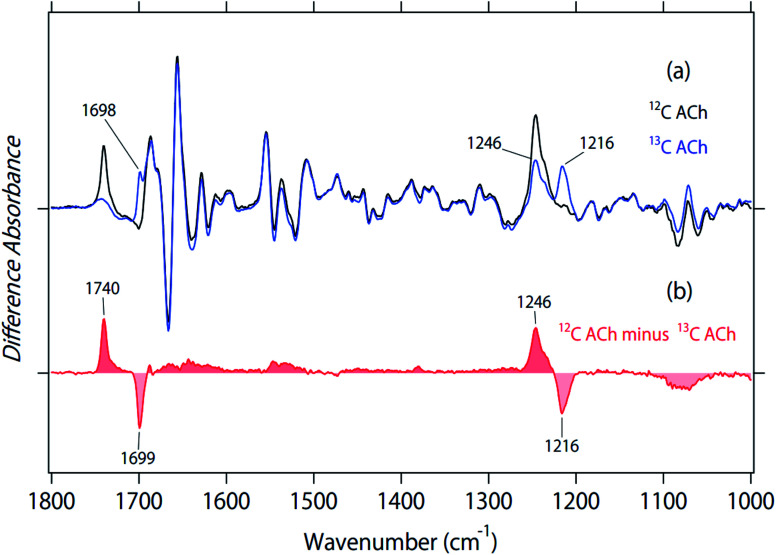
(a) Difference ATR-FTIR spectra upon binding of acetylcholine to M_2_R. Black and blue lines are the spectra for unlabeled and 2-^13^C-labeled acetylcholine, respectively. Positive and negative bands originate from either ligand-bound active or ligand-free inactive states, respectively. One division of the *y*-axis corresponds to 0.0017 absorbance units. (b) The double difference spectrum of (a), in which the blue spectrum is subtracted from the black spectrum.

Black and blue spectra in Fig. 2a represent the binding of unlabeled and 2-^13^C-labeled ACh to M_2_R, respectively. The spectral contributions of the unbound ligand, protein/lipid shrinkage, and water/buffer components were corrected as described previously.^18^ Positive and negative signals originate from the ACh-bound and free receptors, respectively. Therefore, only positive bands contain signals owing to ACh. A positive peak at 1741 cm^−1^ of the black spectrum in Fig. 2a disappears in the blue spectrum, while a positive peak newly appears at 1698 cm^−1^ in the blue spectrum. The isotope shift is shown more clearly in the double difference spectrum (^12^C minus 2-^13^C spectrum) in Fig. 2b, and the spectral shift by 41 cm^−1^ is coincident with the calculated value using a reduced mass for the ^12^C=O and ^13^C=O groups (39 cm^−1^). Thus, the band at 1740 cm^−1^ was assigned as the C=O stretch of ACh.

The 1800–1700 cm^−1^ region is characteristic of protonated carboxylic acids, although the frequencies for deprotonated carboxylic acids (1650–1550 cm^−1^ and 1450–1350 cm^−1^) overlap with other vibrations and their identification is difficult. D103^3.32^ is probably located at the position in contact with ACh in the bound M_2_R, as is iperoxo (Fig. 1a). As ACh is positively charged, D103^3.32^ is likely to be deprotonated for the charge balance. In contrast, the protonation state of D103^3.32^ is unclear in the ligand-free M_2_R. If D103^3.32^ is protonated in the free form, the bound-minus-free difference FTIR spectra should exhibit a negative band at 1800–1700 cm^−1^. The 1741 cm^−1^ band was shifted to 1698 cm^−1^, while a small positive band remained at about 1750–1730 cm^−1^ for the 2-^13^C-labeled ACh (blue line in Fig. 2a). This possibly originates from protonated carboxylic acid, which will be examined by the measurement in D_2_O below.

Fig. 2a also shows that a positive peak at 1246 cm^−1^ of the black spectrum reduced the amplitude in the blue spectrum by half, while a positive peak newly appeared at 1216 cm^−1^ for the blue spectrum. The isotope shift is clearly shown in the double difference spectrum (^12^C minus 2-^13^C spectrum) in Fig. 2b, and the spectral shift by 30 cm^−1^ coincides with the calculated value using a reduced mass for the ^12^C–O and ^13^C–O groups (28 cm^−1^). Thus, we assigned the band at 1246 cm^−1^ as the C–O stretch of ACh. It should be noted that we also observed a peak at 1246 cm^−1^, whose amplitude is similar to that at 1216 cm^−1^ (blue line in Fig. 2a). This suggests that the band at 1246 cm^−1^ is composed of multiple vibrations, including the C–O stretch of ACh. In addition to the tagged peaks, there were some spectral deviations between black and blue spectra, which is also clear in the double difference spectrum (red curve in Fig. 2b). Nevertheless, no clear positive/negative peak pair was observed.

### Deuterium effect of difference FTIR spectra upon acetylcholine binding

**Fig. 3:**
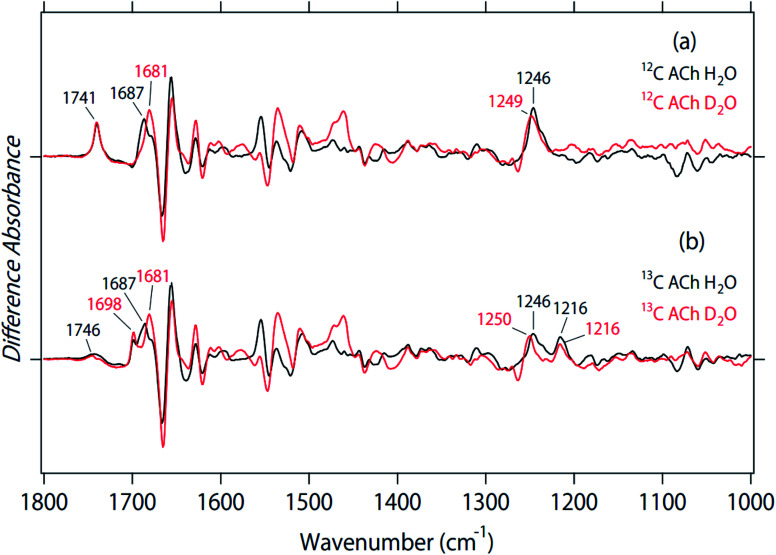
The effect of deuterium on the spectra for unlabeled (a) and 2-^13^C-labeled (b) acetylcholine. Black and red lines are the spectra in H_2_O and D_2_O, respectively.

Fig. 3 shows the effect of deuterium on the ACh-bound M_2_R spectra for unlabeled (a) and 2-^13^C-labeled (b) ACh. The band at 1741 cm^−1^ was insensitive to the H–D exchange (Fig. 3a), which is consistent with the assignment as the C=O stretch of ACh. The inability to exchange H–D was also the case for the ^13^C=O stretch at 1698 cm^−1^ (Fig. 3b), supporting the assignment of the vibration from ACh. As described above, a small positive band at 1746 cm^−1^ and a broad negative band at 1730–1700 cm^−1^ were observed for the ^13^C-ACh bound M_2_R spectra (black line in Fig. 3b), which could originate from the C=O stretch of protonated carboxylic acids. Although the C=O stretch of protonated carboxylate generally demonstrates a down-shift in D_2_O, we did not observe a spectral down-shift for the bands at 1750–1700 cm^−1^ in D_2_O (Fig. 3b). The two spectra in Fig. 3b considerably deviate with each other (expanded in Fig. S1†), but the H/D shift was not observed. Therefore, we conclude that there were no signals of protonated carboxylic acids in the ACh-bound M_2_R spectra. This suggests that D103^3.32^ is deprotonated in both ACh-bound and free states. The observed bands at 1750–1700 cm^−1^ may originate from the C=O stretch of lipids.

In a previous paper, we also studied the binding of atropine to M_2_R.^18^ Unlike positively charged ACh, atropine is a neutral antagonist. From the effect of deuterium on the atropine-bound M_2_R spectra, we suggest the possibility of D103^3.32^ protonation of both bound and free states. However, atropine also contain a C=O group, and it is difficult to identify the protonation state of carboxylic acids in the presence of the C=O ligand. In this sense, the present spectral analysis (Fig. 3b) is more convincing, and we conclude that ligand-free M_2_R contains a negatively charged D103^3.32^.

Previously we tentatively assigned the positive band at 1687 cm^−1^ to the C=O stretch of N404^6.52^, as it exhibited a 6 cm^−1^ down-shift to 1681 cm^−1^ in D_2_O (Fig. 3a).^18^ This spectral feature was also the case for 2-^13^C-labeled ACh (Fig. 3b), which is consistent with our previous interpretation. It should be noted that these peaks diminished in atropine-bound M_2_R spectra.^18^ Therefore, the hydrogen bond of N404^6.52^ with an agonist, but not with an antagonist, plays a crucial role in the formation of an active structure. This view is supported by previous crystal structures of inactive^11^ and active M_2_R^12,13^ as well as previous mutagenesis studies.^12^

The positive 1246 cm^−1^ band for unlabeled ACh exhibited an up-shift to 1249 cm^−1^ in D_2_O (Fig. 3a), suggesting that this band originated from the C–O stretch of a tyrosine residue. However, as can be seen in Fig. 2, the band at 1246 cm^−1^ is split into two peaks for 2-^13^C-labeled ACh. Fig. 3b shows that the positive 1216 cm^−1^ band is unexchangeable H–D, which is consistent with the assignment as the C–O stretch of ACh. On the other hand, the positive 1246 cm^−1^ band was up-shifted to 1250 cm^−1^ in D_2_O. An upward shift is characteristic of the C–O stretch of tyrosine. The amino acid residues, Y104^3.33^, Y426^7.39^, and Y403^6.51^ are part of the hydrogen-bonding network that forms the “tyrosine lid” of the ligand-binding pocket that excludes solvent entry,^4,19^ and site directed mutagenesis of these tyrosine residues leads to impaired agonist binding.^4^ Therefore, the positive band at 1246 cm^−1^ in Fig. 3b probably originates from the tyrosine lid. A corresponding band was not observed in the atropine-bound M_2_R spectrum.^18^

It is known that sodium binding allosterically modulates several GPCRs such as the adenosine receptor,^20,21^ suggesting that physiological concentrations of sodium ions affect functionally relevant conformational states of GPCRs. Here we tested binding of sodium ions to M_2_R. Fig. S2† clearly demonstrate the difference spectra between 140 mM sodium and potassium ions coinciding at the baseline, both in the absence and presence of ACh. This observation excludes sodium binding to M_2_R under the present conditions. It should be noted that one approach alone is not suited for rejecting the hypothesis that sodium might interact with this particular GPCR. However, the present ATR-FTIR spectroscopy was applied to the purified M_2_R protein in lipid membranes, which was gently attached to the IR prism in buffer solution. Therefore, the present *in vitro* measurements are performed under the most physiological conditions. In addition, vibrational signals are enough sensitive to distinguish the sodium binding. We thus safely conclude no sodium binding to the purified M_2_R protein *in vitro*. It is intriguing to test sodium binding to other GPCRs by ATR-FTIR spectroscopy, which will be our future focus.

### FTIR spectra of 2-^13^C acetylcholine in aqueous solution

**Fig. 4:**
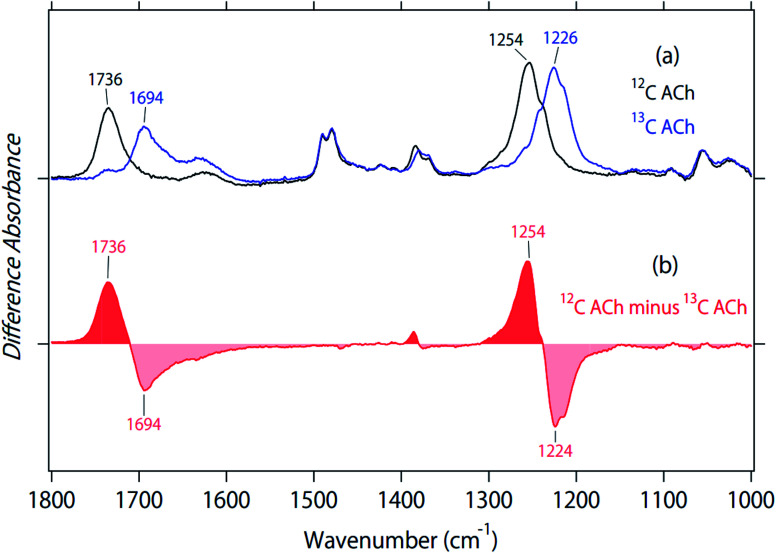
(a) Absorption spectra of unlabeled (black line) and 2-^13^C-labeled (blue line) acetylcholine in aqueous solution. (b) Difference spectrum of (a), in which the blue spectrum is subtracted from the black spectrum.

Fig. 2 and 3 depict structural features of ACh in M_2_R. To investigate the structure in a protein environment, we measured the spectra of unlabeled and 2-^13^C-labeled ACh in aqueous solution. The black line in Fig. 4a represents the absorption spectrum of ACh, and the two strong peaks at 1736 and 1254 cm^−1^ show a spectral down-shift of ^13^C labelling, supporting the interpretation of C=O and C–O stretches of ACh, respectively, in the protein environment (Fig. 2). In aqueous solution, the C=O group of ACh probably acts as the hydrogen-bonding acceptor of water molecules, located at an energetically favourable position. This implies that the hydrogen bond of the C=O group of ACh is very strong in aqueous solution, and that the frequency (1736 cm^−1^) is an indicator of such a strong hydrogen bond. The frequency in M_2_R (1741 cm^−1^) is slightly higher, but is similar to that in aqueous solution.

Previous IR multiphoton dissociation experiments reported three peaks at 1751, 1778, and 1794 cm^−1^ for ACh in the gas phase.^22^ ACh is a flexible molecule, and the terminal C=O group is able to interact with the positively charged terminal in a folded conformation, which probably explains the C=O stretch at 1751 cm^−1^. In contrast, the remaining two bands at 1778 and 1794 cm^−1^ are very high, corresponding to the C=O stretch without any interaction. Therefore, the C=O stretch of ACh in M_2_R (1741 cm^−1^) has a much lower frequency than the free C=O stretch (1778–1794 cm^−1^), but is very close to that in aqueous solution (1736 cm^−1^). One may believe that polar aprotic solvents such as dimethylsulfoxide (DMSO) provide better control than in the gas phase, but it is not the case. Fig. S3† shows that the C=O stretch appears at 1744 cm^−1^ in DMSO. Although DMSO solvent is regarded as no hydrogen-bonding environment, it is likely that Ach forms a folded conformation in DMSO, which enables the terminal C=O group interacting with the positively charged terminal as observed in the gas phase (1751 cm^−1^). These facts indicate that the hydrogen bond of the C=O group in M_2_R is very strong, as strong as in water. We will below propose a structural model based on the present observation.

The C–O stretch appears at 1254 cm^−1^ in aqueous solution, which is 8 cm^−1^ higher than in protein. IR multiphoton dissociation experiments reported three peaks at 1186, 1201, and 1205 cm^−1^ as the C–O stretch of ACh in the gas phase.^22^ Whereas the addition of water molecules in the cluster increased the frequency in gas-phase spectroscopy,^22^ in our study, a comparison among the gas phase, aqueous solution, and protein did not provide important structural information, unlike the C=O stretches. Fig. 4a and b show a small isotope effect at 1400–1350 cm^−1^, which was also observed in Fig. 2b. On the other hand, other vibrations exhibited no isotope effect.

**Fig. 5:**
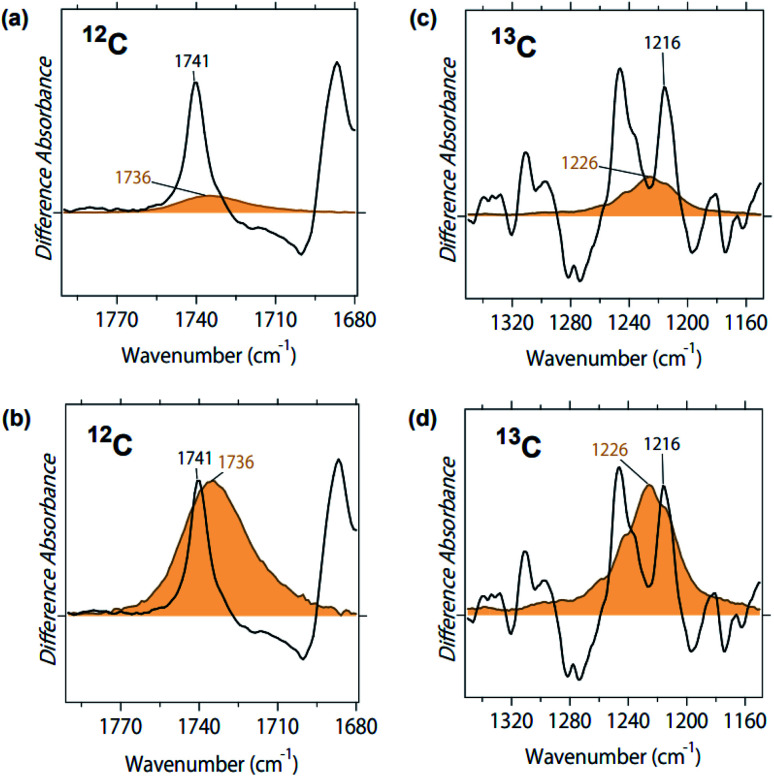
(a) Spectral comparison of the C=O stretch of ACh in M_2_R (black line) and in aqueous solution (orange line). The concentration of ACh is 1 mM in both cases. (b) Normalized peaks of the C=O stretch of ACh in M_2_R (black line) and in aqueous solution (orange line), where the orange band was multiplied by 7.5. (c) Spectral comparison of the C–O stretch of ACh in M_2_R (black line) and in aqueous solution (orange line). Concentration of ACh was 1 mM in both cases. (d) Normalized peaks of the C–O stretch of ACh in M_2_R (black line) and in aqueous solution (orange line), where the orange band was multiplied by 3.3.

Fig. 5a compares the two peaks of the C=O stretch of ACh in protein (black line) and in aqueous solution (orange line). Although the concentration of ACh was identical (1 mM), the black peak is one order of magnitude larger than the orange peak. This is presumably because of the increased concentration of the former, as M_2_R molecules are attached at the surface of the ATR crystal, to which ACh is specifically bound. Fig. 5b is the normalized spectrum, where the orange band was multiplied by 7.5. As is clearly seen in Fig. 5b, band width was much broader in solution (full width of half maximum (FWHM): 17 cm^−1^) than in protein (FWHM: 4.9 cm^−1^). This indicates a more heterogeneous structure of ACh in solution than in protein. This is reasonable since ACh is free in solution, but restricted in the protein environment.

This is also the case for the C–O stretch of ACh, where the peak at 1246 cm^−1^ (Fig. 3) was much larger than the peak at 1254 cm^−1^ (Fig. 4), and the band width was much broader in solution (FWHM: 21 cm^−1^) than in protein (FWHM: 9.3 cm^−1^). Since the 1246 cm^−1^ band contains protein vibrations, we used the spectra of ^13^C-labeled ACh. Fig. 5c compares the two peaks of the ^13^C–O stretch of ACh in protein (black line) and in aqueous solution (orange line). Fig. 5d is the normalized spectrum, where the orange band was multiplied by 3.3. The band is much broader in solution (FWHM: 23 cm^−1^) than in protein (FWHM: 7.0 cm^−1^).

## Discussion

**Fig. 6:**
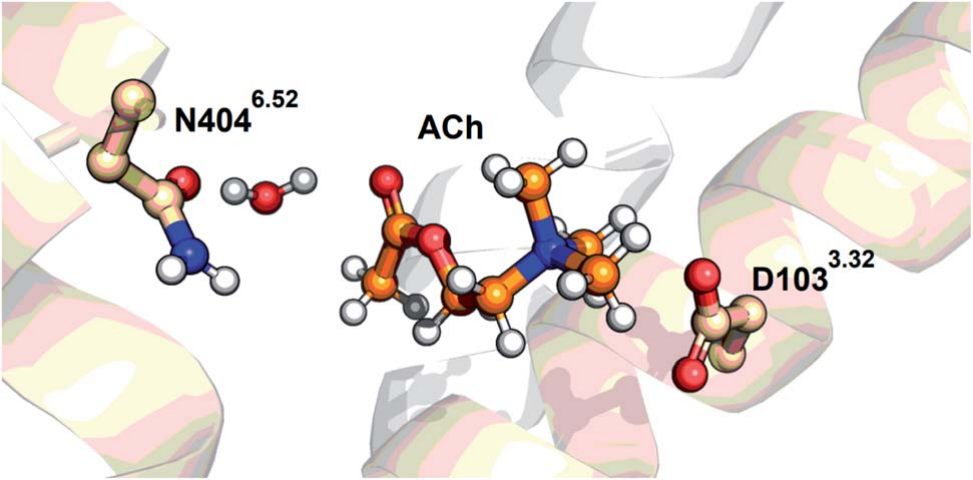
The acetylcholine-docked model in the gauche conformation, obtained based on the crystal structure of the active form of the M_2_R (PDB ID: 4MQS) using Schrödinger suits 2019-3 (Schrödinger, LLC, NY, USA). A water molecule is manually added in the model.

The present FTIR study using 2-^13^C-labeled ACh identified their C=O and C–O stretching vibrations. The C=O stretch of ACh in M_2_R (1741 cm^−1^) is close to that in aqueous solution (1736 cm^−1^), and much lower in frequency than the free C=O stretch (1778–1794 cm^−1^). From the iperoxo-bound M_2_R structure (Fig. 1a), the hydrogen-bonding acceptor is either the N–H group of N404^6.52^ or the O–H group of a water molecule. Here we performed docking simulation of ACh-bound M_2_R based on the crystal structure of the iperoxo-bound form using Schrödinger suits 2019-3, where we fixed the gauche conformation for Ach and added a water molecule. It is likely that a water molecule bridges ACh and N404^6.52^, as shown in Fig. 6. Then, the water molecule possibly forms a hydrogen bond with the N–H or C=O group of N404^6.52^. Our docking simulation suggests that the C=O group forms a hydrogen bond more stably with water than the N–H group. Therefore, we propose a structural model in Fig. 6. It should be noted that this model uses the protein structure of iperoxo-bound M_2_R, and we never calculated an energetically minimized structure for the whole protein. While the ACh-bound M_2_R structure will be studied experimentally and theoretically in the future, we discuss the present FTIR observation using the model structure in Fig. 6.

The H/D-sensitive 1687 cm^−1^ band (+) in Fig. 3 is most likely attributable to the C=O stretch of N404^6.52^. Note that the corresponding negative band is absent. As the C=O stretch of N404^6.52^ must be present in the free form, the signal amplitude of the C=O stretch is intensified upon ligand binding. In the model structure of Fig. 6, we propose that the C=O group of N404^6.52^ is the hydrogen-bonding acceptor of the bridged water, which also forms a hydrogen bond with the C=O group of ACh. Such a strong hydrogen-bonding interaction between the ligand and TM6 yields a specific structure at the intracellular surface, leading to the activation of G-protein. We reported that the band at 1687 cm^−1^ diminished in the antagonist atropine-bound M_2_R spectra, suggesting the crucial structural role for forming the active structure, a view that is fully consistent with previous structural and functional studies.^11–13^

The other terminal of ACh is positively charged (Fig. 1d), which probably interacts with negatively charged D103^3.32^. D103^3.32^ is a key residue in M_2_R, whose protonation state is of great interest as it is highly conserved.^23,24^ Although FTIR spectroscopy is good at monitoring protonated carboxylic acids, spectral overlap of the C=O stretch in ACh was problematic. By using 2-^13^C-labeled ACh, in this study, we were able to identify vibrations of protonated carboxylic acids. Interestingly, the present study concluded that D103^3.32^ is deprotonated (negatively charged) in both ACh-bound and free states. It is well known that the pKa of carboxylates increases in the hydrophobic protein interior.^25,26^ There are no positive charges nearby D103^3.32^, and there must be a mechanism to stabilize the negative charge of D103^3.32^ in the free M_2_R. One possibility is that the water-containing hydrogen-bonding network stabilizes the negative charge at D103^3.32^, and that the negatively charged D103^3.32^ would be the driving force behind the binding of positively charged ACh. ACh is a flexible molecule, but there are strong interactions at both termini, one is a hydrogen bond with N404^6.52^ and another is an electrostatic interaction with D103^3.32^, stabilizing the extended conformation of ACh in the gauche conformation.

The present 2-^13^C-labeled ACh study also shed light on the “tyrosine lid”. The positive 1246 cm^−1^ band in Fig. 3b was up-shifted to 1250 cm^−1^ in D_2_O, and the up-shifted nature is characteristic of the C–O stretch of tyrosine.^27^ Therefore, the signal originates from the vibration of tyrosines, presumably from the “tyrosine lid”. Note that the C–O stretch of the tyrosine lid exists in the free M_2_R, but only the positive band was observed, as well as the C=O stretch of N404^6.52^. The reason is probably the same between N404^6.52^ and the tyrosine lid, namely that specific interactions intensify the IR absorbance of each. The positive signal of the tyrosine lid was absent in atropine-bound M_2_R spectra,^18^ as was the C=O stretch of N404^6.52^, suggesting their important role in signal transduction. Although we did not show the tyrosine lid in Fig. 6, these tyrosines must be located in contact with ACh, similar to iperoxo (Fig. 1a). The gauche conformation of ACh is probably stabilized by the tyrosine lid. Since this vibration is known as an indicator of the degree of the proton-donating state in the hydrogen bond on the phenolic O–H group,^28^ detailed analysis will provide further insight about the interaction of the tyrosine lid in future.

The present study successfully identified the C=O and C–O stretches of ACh. Structural information was obtained not only for ACh, but also for protein, including vibrations of side chains such as N404^6.52^, D103^3.32^ and the tyrosine lid. These side chains contribute to the restricted environment of ACh in M_2_R, which differs from the free structure and enables the activation of G-protein. Such structural differences between ACh-bound and free M_2_R were most significantly seen in the amide-I region at 1670–1650 cm^−1^ (Fig. 1b). In fact, the peaks at 1666 (−)/1656 (+) cm^−1^ are largest in the mid-IR region, and their frequencies are attributable to the α-helix. Helical structural perturbation is a key issue to understand the molecular mechanism of G-protein activation by GPCR,^15,29,30^ where active M_2_R structure shows a large helical displacement of TM6. We previously reported agonist- and antagonist-specific amide-I spectra,^18^ and the functional correlation of amide-I signals is intriguing. The detailed investigation with various ligands is in progress. Structural changes of M_2_R activation may also involve structural changes of lipids, as shown in the H/D insensitive bands at 1750–1700 cm^−1^ (Fig. 3b). In contrast, M_2_R did not bind sodium ions both in the free and ACh-bound forms (Fig. S2†). The structural insight obtained in the present study will lead to a better understanding of the activation mechanism of M_2_R, which will be utilized for drug development. In addition, accurate measurements for the entire mid-IR region (4000 to 1000 cm^−1^) were a real challenge in ATR-FTIR spectroscopy of biomolecules, but we successfully obtained the difference ATR-FTIR spectra upon sodium binding to a rhodopsin KR2 in aqueous solution.^31^ As the frequency at 4000 to 3000 cm^−1^ provide rich information about hydrogen bonds, the measurement for ligand binding to M_2_R is out future focus.

## Materials and methods

### ^13^C-labeled ligand preparation

Acetic anhydride-1,1′-^13^C2 (99 atom% ^13^C, 93.3 μL, 0.987 mmol, 2.0 equiv.) and pyridine (0.199 mL, 2.47 mmol, 5.0 equiv.) was added to a solution of 2-(dimethylamino)ethanol (44.0 mg, 0.494 mmol) in CH_2_Cl_2_ (4.9 mL) at 25 °C, and the mixture was stirred at 25 °C for 24 h. Solvent was removed under reduced pressure and the resulting mixture was co-evaporated with toluene. The resulting solid was diluted with water, and the mixture was basified by 1 M NaOH. The mixture was extracted with CH_2_Cl_2_ and the combined organic phase was washed with brine, then dried with Na_2_SO_4_. Solvent was removed under reduced pressure (>30 torr) to give the 2-(dimethylamino)ethyl acetate-1-^13^C (64.6 mg, quant) as a colorless oil, which was used in the next reaction without further purification. Methyl iodide (0.147 mL, 2.368 mmol, 5.0 equiv.) was added to a solution of 2-(dimethylamino)ethyl acetate-1-^13^C (62.6 mg, 0.474 mmol) in CH_2_Cl_2_ (4.7 mL) at 0 °C, and the mixture was stirred at rt for 24 h. Solvent was removed under reduced pressure to give a solid, which was washed with Et_2_O, then dried under reduced pressure to give acetylcholine iodide-1-^13^C (69.6 mg, quant) as a white solid. ^1^H-NMR (500 MHz, DMSO-*d*_6_) *δ* 4.42 (br s, 2H), 3.66–3.68 (m, 2H), 3.14 (s, 9H), 2.05 (d, *J* = 7.0 Hz, 3H) ppm. ^13^C-NMR (126 MHz, DMSO-*d*_6_) *δ* 169.93, 63.67, 57.76 (d, *J* = 1.8 Hz) 52.93, 20.81 (d, *J* = 59.0 Hz) ppm. HRMS (ESI) *m*/*z*: [M]^+^ calcd for C_6_^13^CH_16_O_2_N 147.1215; found: 147.1210.

### Protein expression, purification and reconstitution

M_2_R fused with BRIL at the ICL3 position (M_2_R) was expressed and purified as described previously.^13^ Briefly, C-terminally His-tagged M_2_-BRIL with the hemagglutinin (HA) signal sequence followed by an N-terminal FLAG tag was expressed in Sf9 insect cells. Cells were infected at a density of 3–4 × 10^6^ cells per mL and grown for 48 h at 27 °C. Sf9 cells were lysed by osmotic shock in the presence of 10 μM atropine (Sigma-Aldrich). The lysed membranes were solubilized with a buffer of 30 mM HEPES-NaOH (pH 7.5), 0.75 M NaCl, 5 mM imidazole, 1% (w/v) *n*-dodecyl-β-d-maltopyranoside (DDM; anatrace), 0.2% sodium cholate (Wako), 1 mg mL^−1^ iodoacetamide (Dojindo) and Complete Protease inhibitor (Roche) for 1 h at 4 °C. The supernatant was isolated by ultracentrifugation for 30 min at 140 000×*g* and incubated with Ni-NTA Sepharose Superflow resin (Qiagen) overnight at 4 °C. After binding, the resin was washed with Ni-NTA wash buffer: 30 mM HEPES-NaOH (pH 7.5), 0.75 M NaCl, 0.1% (w/v) DDM, 0.02% (w/v) sodium cholate, 5 mM imidazole and 10 μM atropin. The protein was then eluted with Ni-NTA elution buffer: 30 mM HEPES-NaOH (pH 7.5), 0.75 M NaCl, 0.1% (w/v) DDM, 0.02% (w/v) sodium cholate, 5 mM imidazole and 10 μM atropine and 500 mM imidazole. The eluate was supplemented with 2 mM calcium chloride and loaded onto an anti-FLAG M1 affinity resin (Sigma-Aldrich). The receptor was eluted from the anti-FLAG M1 affinity resin with a buffer of 20 mM HEPES-NaOH (pH 7.5), 0.1 M NaCl, 0.01% (w/v) DDM, 10 μM atropine, 0.2 mg mL^−1^ FLAG peptide and 5 mM EDTA. Finally, protein was purified by a Superdex 200 Increase size exclusion column (GE Healthcare) in a buffer of 20 mM HEPES-NaOH (pH 7.5), 0.1 M NaCl and 0.01% (w/v) DDM.

For ATR-FTIR measurements, detergent-solubilized M_2_R was reconstituted into asolectin liposomes with a 20-fold molar excess, whose lipid/protein ratio is considerably low. However, detergent-solubilized M_2_R contains certain amount of lipids, and the real lipid/protein ratio is higher, though it was difficult to quantitatively estimate in the present study. The detergent molecule was removed by incubation with Biobeads SM-2 (Bio-Rad). After removing the Biobeads, the lipid-reconstituted M_2_R was collected by ultracentrifugation. After several cycles of wash/spin, lipid-reconstituted M_2_R was suspended in a buffer composed of 5 mM phosphate (pH 7.5) and 10 mM KCl.

### Measurement of ligand binding-induced difference ATR-FTIR spectroscopy

A 2 μL aliquot of the lipid-reconstituted M_2_R suspensions was placed on the surface of a silicon ATR crystal (three internal reflections). After it was dried gently by natural drying, the sample was rehydrated in a solvent containing 200 mM phosphate (pH 7.5) buffer with 140 mM NaCl and 3 mM MgCl_2_ at a flow rate of 0.6 mL min^−1^ through a flow cell whose temperature was maintained at 20 °C by circulating water. ATR-FTIR spectra were first recorded at 2 cm^−1^ resolution using an FTIR spectrometer (Bio-rad FTS7000, Agilent, CA, USA) equipped with a liquid nitrogen-cooled MCT detector (average of 768 interferograms). After the FTIR spectrum had been recorded in the second buffer with 1 mM ligand, the difference FTIR spectrum was calculated by subtracting the data obtained for the first and second buffer. The cycling procedure was repeated two to seven times, and the difference spectra were calculated as the average of the presence minus absence spectra of ligand. The spectral contributions of the unbound ligand, protein/lipid shrinkage, and water/buffer components were corrected as described previously.^18^ In order to avoid a non-specific signal, we consider the signal before circulating the buffer (with ligand) and after circulating the buffer (without ligand), confirming that the signal returned to the baseline. For the measurements in D_2_O media, perfusion buffers with the same composition were prepared using deuterium oxide (99 atom% D, Sigma-Aldrich) instead of deionized water, which was adjusted at pD 7.5, assuming the pD value equals pH_meter reading_ + 0.4.^32^

### Docking simulation of M_2_R and ACh

All calculations were conducted under the OPLS3e force field and performed using Schrödinger suits 2019-3 (Schrödinger, LLC, NY, USA). At first, two conformations of gauche-formed acetylcholine were calculated by only performing energy-minimization. The crystal structure of the active form of M_2_R (PDB ID: 4QMS) was prepared and treated using a protein preparation tool. Next, using the Glide program, two conformations of gauche-formed acetylcholine were docked rigidly to the same place with iperoxo in 4MQS (amine group of acetylcholine was constrained to the amine group of iperoxo).

To consider the effect of a hydrogen bond *via* water, we added a water molecule in the models obtained above. Then, with the Prime program, induced-fit was calculated against the water molecule by constraining the receptor. Since two conformations of acetylcholine were redocked to obtain two models, we obtained four gauche-formed acetylcholine-docked models. Finally, we selected the one that showed the best docking score as the acetylcholine-docked model.

## Author contributions

K. K., R. S., S. I., T. K., N. S. and H. K contributed to the study design. Y. S. prepared isotope-labelled ligand. R. S. and H. T. expressed samples in Sf9 and purified them. K. S. and K. K. reconstituted samples for spectroscopic measurements, and conducted ATR-FTIR spectroscopic measurements. T. N. performed docking simulation. H. K. wrote the manuscript. All authors discussed and commented on the manuscript.

## Conflicts of interest

The authors declare no competing financial interests.

## Supplementary Material

RA-011-D1RA01030A-s001
