# Supplementary material for: Vibrational analysis of acetylcholine binding to the M2 receptor
Source: RSC Adv. 2021 Apr 7;11(21):12559–67. doi: 10.1039/d1ra01030a (PMC8696876; doi:10.1039/d1ra01030a)
Supplement: RA-011-D1RA01030A-s001 [file RA-011-D1RA01030A-s001.pdf]

Electronic Supplementary Material (ESI) for RSC Advances.
This journal is © The Royal Society of Chemistry 2021

Supplementary Information

Vibrational Analysis of Acetylcholine Binding to the M_2 Receptor

Kohei Suzuki[a], Kota Katayama[a,b], Yuji Sumii[a], Tomoya Nakagita[d], Ryoji Suno[c], Hirokazu Tsujimoto[d], So Iwata[d], Takuya Kobayashi[c,e], Norio Shibata[a], Hideki Kandori[a,b,*]

[a] Department of Life Science and Applied Chemistry, Nagoya Institute of Technology, Showa-ku, Nagoya 466-8555, Japan
[b] OptoBioTechnology Research Center, Nagoya Institute of Technology, Showa-ku, Nagoya 466-8555, Japan
[c] Department of Medical Chemistry, Kansai Medical University, Hirakata 573-1010, Japan
[d] Department of Cell Biology, Graduate School of Medicine, Kyoto University, Kyoto 606-8501, Japan
[e] Japan Agency for Medical Research and Development, Core Research for Evolutional Science and Technology (AMED-CREST), Tokyo 100-0004, Japan

*Correspondence to: kandori@nitech.ac.jp

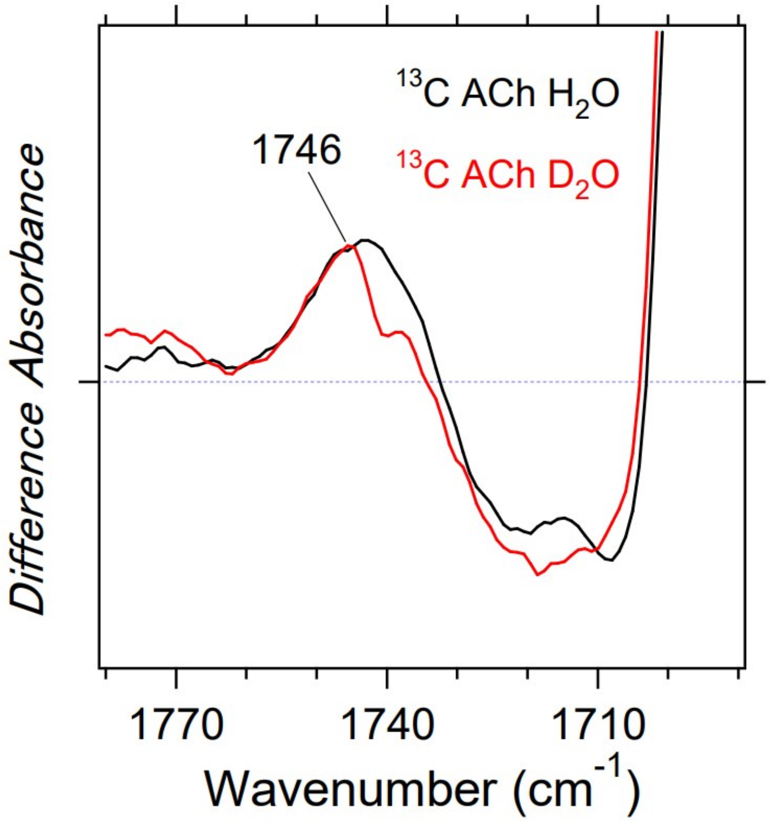

Figure S1. Difference ATR-FTIR spectra upon binding of 2-^{13}C-labeled acetylcholine to M_2R. Black and red lines are the spectra in H_2O and D_2O, respectively. This figure is expanded from Figure 3b.

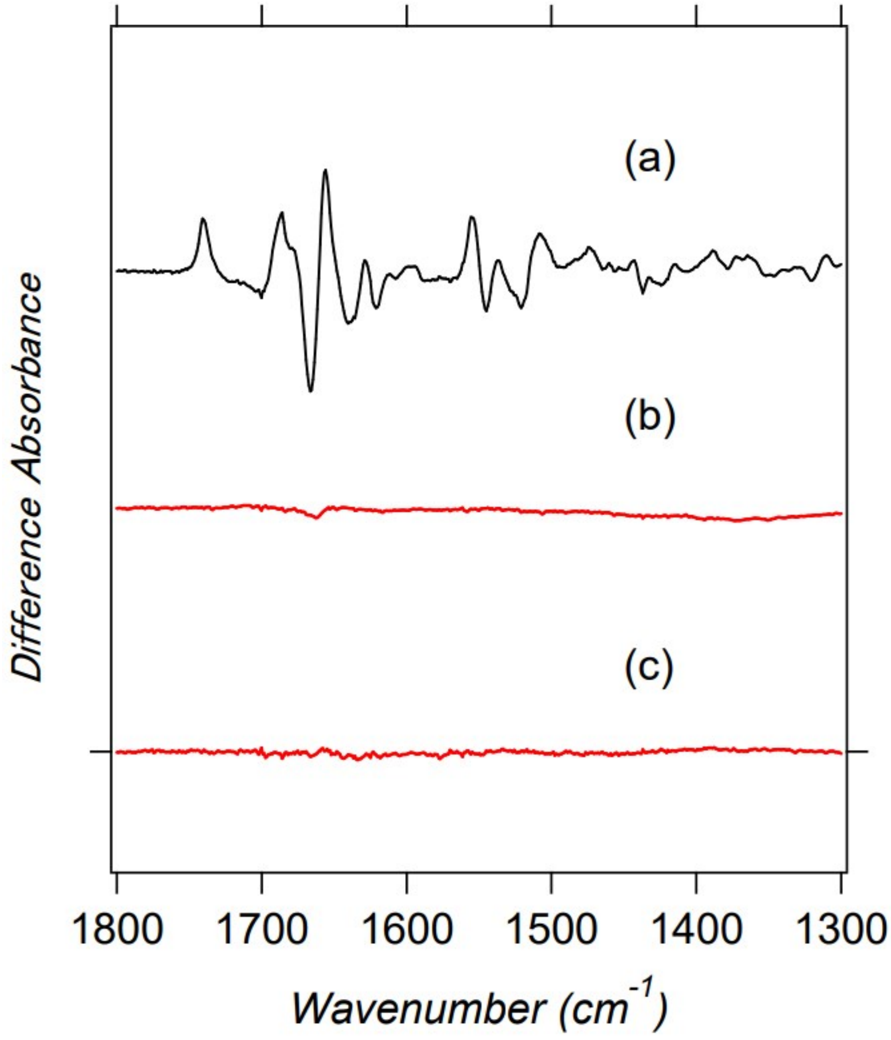

Figure S2. (a) Difference ATR-FTIR spectrum upon binding of acetylcholine (1 mM) to M-$_2$R, where concentration of NaCl is 140 mM. (b) Difference ATR-FTIR spectrum of M_2R between NaCl and KCl (140 mM) in the absence of acetylcholine. (c) Difference ATR-FTIR spectrum of M_2R between NaCl and KCl (140 mM) in the presence of acetylcholine (1 mM).

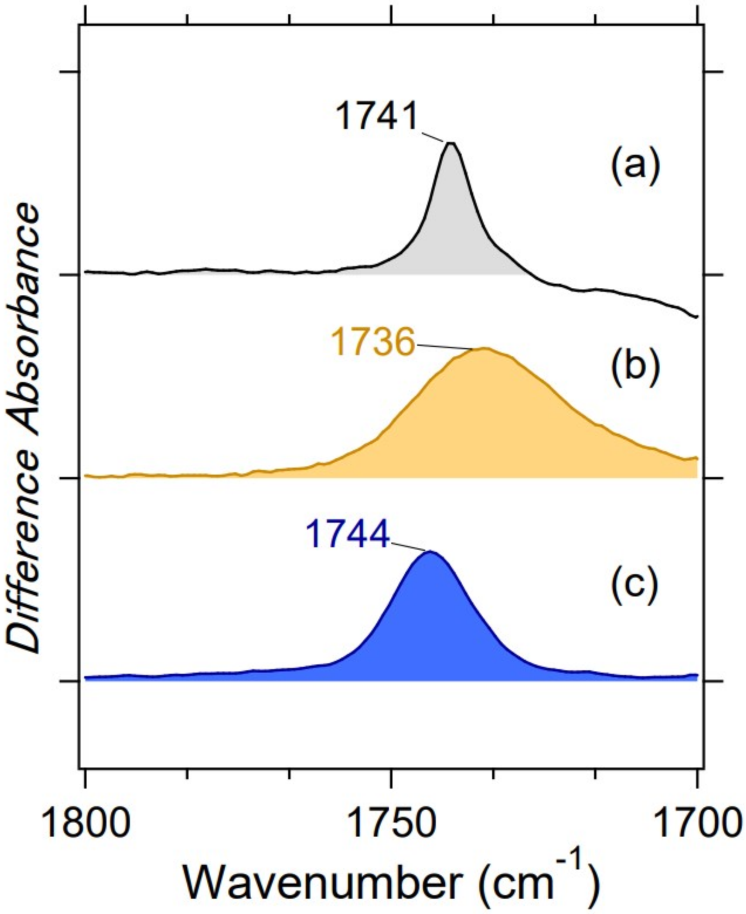

Figure S3. Spectral comparison of the C=O stretch of ACh in M_2R (a), in aqueous solution (b), and in dimethylsulfoxide (DMSO) (c), where peak absorbances are normalized. (a) and (b) are reproduced from Figure 5b. In the gas phase, the C=O stretch of Ach is reported at 1751, 1778, and 1794 cm^{-1},[22] suggesting that the C=O stretches with and without hydrogen bond appear at 1750-1730 cm^{-1} and 1795-1775 cm^{-1}, respectively.
